# Are mild head injuries as mild as we think? Neurobehavioral concomitants of chronic post-concussion syndrome

**DOI:** 10.1186/1471-2377-6-7

**Published:** 2006-02-06

**Authors:** Annette Sterr, Katherine A Herron, Chantal Hayward, Daniela Montaldi

**Affiliations:** 1 School of Human Sciences, University of Surrey, Guildford, UK; 2 School of Psychological Sciences, University of Manchester, Manchester, UK

## Abstract

**Background:**

Mild traumatic brain injury (MTBI) can sometimes lead to persistent postconcussion symptoms. One well accepted hypothesis claims that chronic PCS has a neural origin, and is related to neurobehavioral deficits. But the evidence is not conclusive. In the attempt to characterise chronic MTBI consequences, the present experiment used a group comparison design, which contrasted persons (a) with MTBI and PCS, (b) MTBI without PCS, and (c) matched controls. We predicted that participants who have experienced MTBI but show no signs of PCS would perform similar to controls. At the same time, a subgroup of MTBI participants would show PCS symptoms and only these volunteers would have poorer cognitive performance. Thereby, the performance deficits should be most noticeable in participants with highest PCS severity.

**Method:**

38 patients with a single MTBI that had occurred at least 12 month prior to testing, and 38 matched controls, participated in the experiment. A combination of questionnaires and neuropsychological test batteries were used to assess the extent of PCS and related deficits in neurobehavioral performance.

**Results:**

11 out of 38 MTBI participants (29%) were found to suffer from PCS. This subgroup of MTBI patients performed poorly on neuropsychological test batteries. Thereby, a correlation was found between PCS symptom severity and test performance suggesting that participants with more pronounced PCS symptoms performed worse in cognitive tasks. In contrast, MTBI patients with no PCS showed performed similar to matched control. We further found that loss of consciousness, a key criterion for PCS diagnosis, was not predictive of sustained PCS.

**Conclusion:**

The results support the idea that MTBI can have sustained consequences, and that the subjectively experienced symptoms and difficulties in everyday situations are related to objectively measurable parameters in neurocognitive function.

## Background

Mild traumatic brain injury (MTBI) represents 70 - 90% of all treated brain injuries. It is by far most common in teenagers and young adults, and typically caused by falls and motor-vehicle collisions. The estimated population-based incident rate ranges above 600/100000 [1].

MTBI typically induces a range of symptoms such as: headaches, blurred vision, poor concentration, sleep disturbance, depressed mood or irritability. These post-injury effects are referred to as Post-Concussion Syndrome (PCS), a *transient *condition which is thought to reflect a fully recoverable disturbance of neural function [2-5]. However, long beyond the typical recovery interval of one to three months, at least 15% of persons with a history of MTBI continue to see their GPs because of persistent problems [6-10]. The clinical validity of these sequeale is not undisputed, in part because there is no readily evident physiological damage or deficit that could be made accountable. Consequently, a range of alternative explanations such as involvement in litigation [1] and pre - morbid psychological problems [11] are discussed. In addition, one may question the correctness of the assumption that MTBI is fully recoverable in all cases.

The view that MTBI leads to transient disturbances only is further supported by the absence of structural brain damage in diagnostic MRI images acquired with standard recording sequences[12]. However, the combination of high resolution MRI with specifically tailored scanning protocols provides evidence for microstructural abnormalities in MTBI patients [13-15]. Ultra-structural studies further indicate that MTBI may damage the structure of neurofilaments and cause traumatic axonal injury [16,17]. Together, these findings support the idea that MTBI can introduce primary structural damage in reaction to the axon misalignment, disconnection and swelling that occurs in response to the physical forces inflicted on the neural tissue [17-19]. It therefore appears plausible to assume that microstructural damage can persist, and that this tissue damage may form the pathophysiological foundation of the persisting sequeale some MTBI patients' experience. This assumption gives rise to the hypotheses that the neural damage affects information processing, and henceforth the prediction that only those MTBI patients who suffer chronic PCS symptoms show neurobehavioral deficits.

This idea is not new and various research groups have studied the role of neurobehavioral performance in PCS. However, the existing data do not provide a coherent picture as to whether cognitive performance in longterm MTBI patients is disturbed or not, and if, to what extent symptoms and performance are related. While some studies report a lack of evidence for sustained postconcussive effects of MTBI [20,21], others report general cognitive deficits [22-24], as well as impairments in specific information processing domains such as attention [25,27], working memory [26,27], and processing speed [28]. Two meta-analyses highlight this empirical controversy and come to the conclusion that the relationship between PCS and cognitive impairment is 'generally weak' [29,30].

We propose that the inconsistent findings on the relationship of neurobehavioural performance and PCS might, in part, be due to the fact that most experiments investigated long-term MTBI consequences by studying a cohort of MTBI participants without taking the status of PCS specifically into account. However, it is clear that only a subgroup of MTBI patients continue to report PCS symptoms, and hence a separate assessment of MTBI patients with and without sustained PCS should provide a further insight. The incoherent picture emerging from the literature represents a serious problem for both suffers and health care providers. For example, little diagnostic advice is provided for clinicians regarding the classification and prognosis of PCS, and ICD-10 suggests guidelines for research purposes only. Furthermore, the chronic effects of MTBI compromise quality of life and general well being [30], and given the high incident rate of MTBI, particularly amongst young adults, the socio-economic consequences are drastic [2]. Henceforth there is a need to characterise and understand chronic MTBI effects. In this context, the present experiment studied cognitive performance in a cohort of MTBI patients at least 12 month post-incident, whereby those with and without PCS were studied separately. To control for potential confounding factors, all participants were in employment or studying at University and were not involved in any litigation.

The Rivermead Post-Concussion Symptoms Questionnaire (RPQ) [31] was used to categorise MTBI patients as those with PCS (PCS^+^) and those without PCS (PCS^-^). Thereby the categorisation criterion was based on ICD-10 guidelines. The RPQ contains a list of symptoms commonly associated with PCS and participants are asked to rate the severity of each item on a scale from 0 - 4. Day-to-day cognitive function/dysfunction was further measured with the Cognitive Failures Questionnaire (CFQ) [32]. The rationale for employing the CFQ as an additional diagnostic tool was based on findings, which suggest that disruptions of daily activities represent a significant correlate of the PCS symptomatic [33-35]. To establish the relationship between subjectively experienced deficits and objectively measurable indices, we further employed a series of neurobehavioral measures. Based on the assumption that frontal lobe functions play a crucial role in modulating information processing and cognition, as well as on previous evidence suggesting deficits in these domains [2,25], the Test of Attentional Performance (TAP) [36] and the Cambridge Neuropsychological Test Automated Battery *expedio*, (CANTABe) [37] were used. The principle study design was based on the rationale that only a sub-group of MTBI patients would have contained sustained PCS, presumably because of persistent microstructural damage. We therefore predict that MTBI patients with PCS would have lower performance scores on the objective measures than those without PCS and the control group. We further hypothesize that PCS severity, indexed by the RPQ sum score and the CFQ, would correlate with the performance indices reaction time and error rate.

## Method

### Participants

Thirty eight head injured and 38 healthy control participants, with a mean age of 23.8 and 23.1 years respectively, were tested. The groups were further matched for handedness, gender and education. Recruitment was conducted through advertisement posters, which invited persons with MTBI as well as healthy controls to contact the laboratory. Posters were distributed in 150 general practitioner surgeries in Merseyside, a local Brain Injury Community Centre, and the University of Liverpool campus. Further information on group characteristics is summarised in table 1.

Participants in the MTBI group were selected on the basis of 'the diagnostic protocol for Mild Traumatic Brain Injury' defined by *The Mild Traumatic Brain Injury Committee of the Head Injury Interdisciplinary Special Interest Group of the American Congress of Rehabilitation Medicine*. Our inclusion criteria for the MTBI cohort comprised: (1) Loss of Consciousness < 30 mins; (2) Post Traumatic Amnesia < 24 hours; (3) alteration of mental state (dazed, disorientated, confused) at time of incident; and (4) one-off MTBI. Most importantly, participants were tested at least 12 months post-incident. Only those who had not been involved in or considered litigation were included in the study. All participants were in employment or studying for at least six month when tested.

Both the MTBI and the control cohort were screened for general well-being, which included questions on mood, depression and anxiety, sleep disturbances, previous psychological or neurological problems, medication, and pain. Those with poor screening outcome were excluded from the experiment. The age range was set to 18–65 years. The study was approved by the South Sefton Research Ethics Committee as well as the Ethics Committee of the Psychology Department at the University of Liverpool, UK. Informed consent was taken prior to participation. The experiment was conducted in a neuropsychological laboratory at the University of Liverpool. Testing took approximately 90 minutes. Participation was reimbursed at £5/hour.

### Group allocation

The RPQ, a 16-item symptom list, which uses a rating scale of 0 to 4 (4 = maximal severity), was employed for the categorisation and the quantification of PCS symptoms. The categorisation was based on the RPQ cut-off criterion (at least three items with severity ratings of 3 or higher, [38]), and resulted in two sub-groups: participants with PCS and participants without. This criterion was satisfied in 11 persons (PCS^+^group) all of whom had suffered MTBI. The criterion for PCS was not met in the remaining 27 participants of the MTBI group (PCS^-^, N = 27) and all control participants (C, N = 38). To quantify the level of PCS severity an RPQ sum score was further calculated for each participant. This allowed us not only to correlate behavioural performance with PCS severity, but also to study our data independent of the group categorisation. The latter is particularly important since the typical PCS symptoms are non-specific and may also be experienced by healthy controls [39]. Therefore analyses based on both, PCS categorisation and overall PCS symptom severity, should enhance the interpretability of the acquired data.

### Measures

Subjective measures comprised the RPQ and the CFQ, a self-rating instrument designed to assess cognitive failures in perception, memory and motor function experienced in every-day life situations. It consists of 25 items which are rated for their frequency of occurrence from 0 to 4 (4 = very often).

Cognitive functioning and performance was measured quantitatively with the two computer-based test batteries, TAP and CANTABe. These tests are specifically designed to assess differential deficits in frontal lobe function. They involve time/accuracy tasks of varying complexity, which are presented in several subtests. In the TAP the response mode contains a simple button press. The CANTABe uses a touch screen system with a response pen. In both tests, the response was exercised with the dominant hand.

From the TAP, the five subtests: *Alertness (AL)*, *Working Memory (WM)*, *Divided Attention (DA)*, *Go/No go (GN)*, and *Covert Shift of Attention (CS)*, were selected to assess deficits in sustained attention, working memory, divided attention, selective attention and switching attention, respectively. From the CANTABe the subsets: *Big Little Circle *(BLC), *Spatial Working Memory *(SWM), *Intra-Extra Dimensional Set Shifting *(IED), *Reaction Time *(RTI) and *Rapid Visual Information Processing *(RVP) assessing shifting/selective attention, working memory, shifting attention, sustained attention with a motor component and selective attention respectively. In addition we employed the National Adult Reading Test [40] to obtain an estimate of the participants' IQ.

### Procedure

The assessment was conducted in a fully controlled environment. Informed consent was taken prior to testing. Data collection was split into two sections, completion of questionnaires and administration of test batteries. The order of sections and the order of tests within sections was quasi-randomised and followed eight counterbalanced orders. The NART data was collected after section one. Depending on the individual's needs, short rest periods were interspersed between tests.

### Analyses

Two-way repeated measure Anovas, comprising the factors group (PCS^+^, PCS^-^, C) and subtest (AL, WM, DA, GN, CS, BLC, SWM, IED, RTI, RVP), were calculated for reaction times and error rates, respectively. Post-hoc group differences for individual subtests were assessed with unpaired t-tests. The relationship of test performance and PCS symptom severity was tested by calculating Pearson correlations between RPQ scores and reaction times/error rates for each subtest. Standard statistics analysis software (SPSS 11.0, StatView 5.0.1) was used for all calculations.

## Results

### Demographical analyses

Analysis of demographic variables and NART scores revealed no significant differences between groups. We further found that injury-related parameters, i.e. chronicity, loss of consciousness or hospitalisation, did not differ significantly between PCS^+^/PCS^- ^groups (Table 1).

### Reporting of PCS symptoms (RPQ & CFQ)

The ANOVA of RPQ sum scores revealed a significant main effect group (F_ [2, 73] _= 15.75, P < .01) which reflected higher RPQ sum scores in the PCS^+ ^group than in the PCS^- ^group (mean difference = -15.1; critical difference: 6.8; P < .01) and the control group (mean difference = 14.5; critical difference: 7.1; P < .01). Most importantly, the difference between the PCS^- ^and controls was insignificant. A similar result pattern was found for the CFQ. A main effect group (F_ [2,73] _= 10.5, P < .01) indicated that participants in the PCS^+ ^group experienced significantly more cognitive failures in their everyday life than controls (mean difference = -7.5, critical difference = 4; P < .01) and PCS^- ^(mean difference: 5.5, critical difference = 4.2; P < .01). Again, the post-hoc contrast between controls and the PCS^- ^group was insignificant.

**Figure 1:**
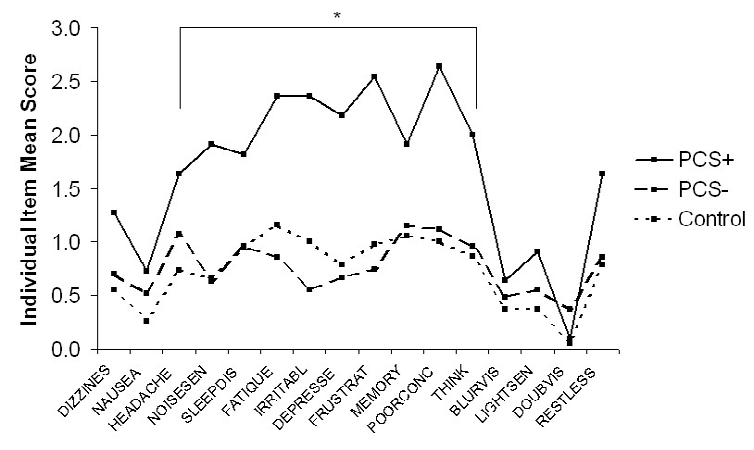
Group means for individual RPQ items. The figure illustrates that RPQ ratings are generally higher for PCS^+ ^than for PCS-and Controls.

An item x group interaction (F_[30, 1095] _= 2.9, P < .01) further indicated that the majority of symptoms were experienced significantly more often in the PCS^+ ^group. Post-hoc analysis of the latter revealed no group differences for the items: restlessness, double vision, light sensation, blurred vision, nausea, and dizziness, while all other RPQ symptoms were experienced significantly more frequently in the PCS^+ ^than the two other groups (PCS-/controls). The effect is illustrated in figure 1.

### Neurobehavioral test data (TAP and CANTABe)

**Figure 2:**
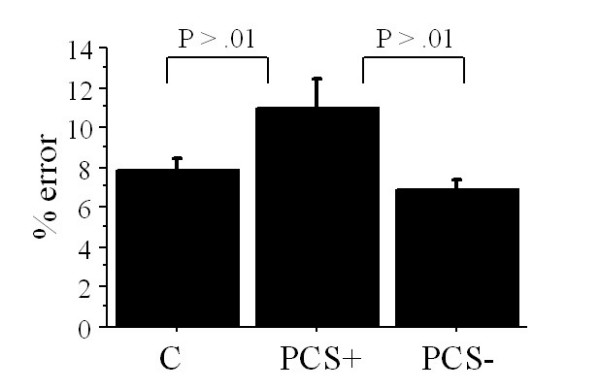
Main effect group for % erors.

Analysis of error scores revealed a significant group (F_[2, 73] _= 5.0, P <.01) and group x subtest interaction (F_[22, 803] _= 2.6; P < .01, GG corrected). The main effect is depicted in figure 2. Post-hoc analysis of the main effect showed that error rates were significantly higher in the PCS^+ ^group as compared to the PCS^- ^group (mean difference = 4.0, critical difference = 3.1; P < .01) and controls (mean difference = -3.0, critical difference = 3.0; P <.05). Unpaired post-hoc t-tests further revealed significant group differences between PCS^+ ^and PCS^- ^for DA (t_(36) _= 2.6, P <.05), WM (t_(36) _= 3.0; P < .01) and RTI (t_(36) _= 2.2; P < .05), as well as a trend for SWM (t_(36) _= 1.8; P =.09). The Anova of RTs revealed no significant effects.

### Correlation analysis

**Figure 3:**
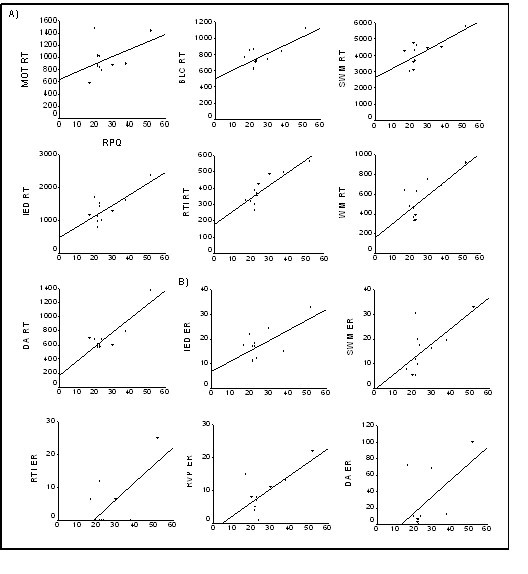
Bivariate scattergrams relating to the significant correlations of symptom severity and performance parameters. Reaction time is depicted in part A, and Error rates are shown in part B. Note that the X axis in each graph represents mean RPQ score.

The correlations of subtest performance with the RPQ sum scores are summarized in table 2. In the PCS^+ ^group, significant correlations were found for 12 out of the 24 subtest scores. In all these cases symptom severity was positively correlated with test performance (table 2 and figure 3). 22 out of the 24 correlations were insignificant in the PCS^- ^group and none of the correlations were significant in controls.

## Discussion

The present experiment was designed to further our understanding of chronic effects following mild head injuries, which remains a largely controversial but equally important public health issue. More specifically, we aimed to characterize sustained PCS and its neurobehavioural concomitants within a group of long term MTBI sufferers. Thereby, a dissociation of PCS symptomatic and MTBI was introduced through group allocation methods. By and large, previous research has failed to produce a clear picture on sustained PCS, mainly because the majority of these experiments did not distinguish between MTBI participants with and without PCS. To this end, the present experiment studied a group of participants who had experienced a one-off MTBI at least twelve months prior to testing, and a matched control group, for PCS. Indexed by the RPQ cut-off criterion, 11 persons (29%) in the MTBI cohort were found to suffer from sustained PCS (PCS^+^), while the remaining 27 persons (71%) showed similar RPQ scores to healthy control participants. In addition, cognitive failures in everyday situations were reported more frequently in the PCS^+ ^group as compared to participants in the PCS^- ^and healthy control groups. These results have two main implications. First, they highlight that mild head injury leads to a chronically elevated level of PCS symptoms in some persons with MTBI, while in others the experienced symptom level is no different from persons who did not have an MTBI. Second, they indicate that neurobehavioral deficits are related to high levels of PCS symptoms but not the MTBI per se. Most importantly, these findings were obtained by using a cut-off criterion in the RPQ for the initial group allocation, but the RPQ sum score, i.e. the complete RPQ symptom profile, for all subsequent analysis.

Our study further revealed interesting results on the role of loss of consciousness in sustained PCS and/or its severity. According to ICD - 10, a 'history of head trauma with loss of consciousness preceding the onset of symptoms by a period of up to four weeks' represents one of the diagnostic criteria (criterion 'B'). However, in our experiment 4 out of 11 participants in the PCS^+ ^group (36%) had not experienced loss of consciousness but nevertheless satisfied all other PCS criteria. At the same time, 14 out of 27 participants (52 %) in the PCS^- ^group had lost consciousness during the incident but did not develop chronic PCS. This is consistent with other studies [41,42], where PCS was found in the absence of loss of consciousness. Most notably in this context, Umile et al. [15] recently reported that participants with PCS performed poorer in neuropsychological tests and showed structural abnormalities in high-resolution MR scans after MTBI without loss of consciousness. Together, these and our findings provide initial evidence that a critical re-evaluation of the loss of consciousness criterion in ICD-10 may be considered. Of course one may argue that our findings on loss of consciousness are of questionable reliability, due to the circumstances under which the majority of incidents occur. However, the patient themselves typically remain the only source of information, henceforth the subjectively perceived loss of consciousness - whether it was truly experienced or not - is probably of limited diagnostic value.

A further aim of the present study was concerned with characterizing potential neurobehavioral correlates of sustained PCS. Based on the hypothesis that mild brain injuries may involve minimal structural damage, we presumed that sustained PCS should be reflected in diminished performance during cognitive tasks. To this end, a correlational analysis of test performance and symptom severity, indexed by the RPQ scores, revealed positive correlations with error rates for 50% of the neurobehavioral subtests in the PCS^+ ^group. Thus, our data clearly suggests that PCS severity is associated with objectively measurable performance deficits in some cognitive tasks. These results highlight the fact that only those MTBI participants who suffer from PCS show deficits in cognitive processing, and provide further evidence that the level of PCS severity and not the experience of MTBI per se is the critical factor. The findings obtained by the correlational analysis have further importance with regards to the often criticized 'unspecific everyday' nature of the RPQ symptom checklist. Studies have shown non-clinical groups to report symptoms similar to those with PCS (e.g. [39]), which led to claims that the RPQ is an unreliable measure for PCS diagnosis (e.g. [43,44]). However, the absence of a systematic relationship between RPQ and cognitive performance indices in the PCS^-^/control groups, but highly correlated scores in the PCS^+^group found in the present study, identify the RPQ as a valid diagnostic measure despite the unspecific nature of checklist items [4].

The assessment of neurocognitive function revealed significantly poorer response accuracy in the PCS^+ ^group than in PCS^- ^and controls, but no systematic effects for reaction time. This leaves three possible explanations. One could either conclude that persisting PCS is unlikely to affect a person's speed of response to stimuli, that RT non-result is due to low test power and thus an effect of sample size, or that participants in the PCS^+ ^group may have greater motivation to perform well in the task. The latter explanation is strongly supported by Potter et al. [24], who suggested that some people with MTBI have heightened anxiety in novel testing situations, because of fears that their performance is affected by their condition. As a result, these participants may be in a state of higher arousal which impacts on their response execution time. For the present study, Potter's argument gains further credibility when error rates are taken into the equation. Thus, participants in the PCS^+ ^group showed higher error rates than participants in both other groups, while reaction times were unaffected. Peloso [44] further argues that the simultaneous management of response accuracy and response speed becomes less efficient when the cognitive load exceeds a certain threshold level, and that this level may be lower following minor brain damage. This idea is well compatible with our finding that the highest error rates were found for the subtests with greatest cognitive demand. Furthermore, cognitive and emotional problems are known to persists longer after the incident than somatic symptoms [45]. In line with this observation, the RPQ symptom profile shows that the experienced symptoms in the PCS^+ ^group relate mostly to cognitive processing, with poor concentration and irritability receiving the highest scores.

A continuing controversy in the discussion of chronic PCS concerns other contributing factors such as malingering [11,46-49]. These concerns are partly driven by the lack of clear evidence for objectively measurable correlates of PCS. We cannot totally rule out the possibility of malingering or other contributing factors to our findings. However, this influence is probably of negligable magnitude for several reasons. First and foremost, participants involved in litigation of any kind were excluded from the study. Secondly, our volunteers were in employment or studying, and generally in good health and spirits. Finally, the severity of PCS symptoms systematically varied with the level of performance across a range cognitive tasks, and such a data distribution across a group is unlikely to be explained by malingering or exaggerated responses in the RPQ.

The research hypotheses of the present paper were based on the assumption that mild head injury can cause permanent microstructural damage and disturbances of neural function. As a result, information processing may become less efficient or more effortful and lead to deficits in neurobehavioral performance. Of course the question regarding the neural origin and mechanisms underlying sustained PCS are not addressed in the present study, however the data is in line with these ideas. Only a subgroup of MTBI participants show PCS symptoms, cognitive failures in everyday situations and diminished neurobehavioural performance in standardized test batteries. This neuroscience-based interpretation of our data might be challenged by the view that sustained PCS is driven by psychological factors rather than structural consequences of brain damage [45,53,54]. For example, Van Zomeren [55] and Wong [56] argued that PCS related symptoms may be manifested to compensate for 'behavioural faults' which patients attribute to the head injury. While this theory could explain why common complaints reported in the PCS^+ ^group are more psychological in origin than somatic, it falls short of a plausible explanation for the strong correlation of objectively measurable performance indices and symptom severity.

In conclusion, the present study demonstrates a link between chronic PCS and neurobehavioral performance with a study design that controlled for the influence of MTBI per se. Our results are in line with some of the literature on long-term consequence of mild head injuries, and particularly highlight the role neuropsychological concomitants. Mild head-injuries are not always to be as mild as the name would suggest, and long-term consequence may very well have a neural underpinning.

## Competing interests

The author(s) declare that they have no competing interests

## Authors' contributions

AS hold overall responsibility for the study. All authors contributed to data collection.

## Pre-publication history

The pre-publication history for this paper can be accessed here:



## Figures and Tables

**Table 1:** Participant Demographics: Age, National Adult Reading Test (NART) and chronicity values expressed as mean (± 1 standard deviation). Loss of consciousness is abbreviated to LOC. All participants have normal eyesight and hearing, and were educated to degree level.

	***PCS *+**	***PCS -***	Controls
**N (76)**	11	27	38
**Age (years)**	24.27 (9.27)	23.33 (3.11)	23.13 (7.94)
**Gender (M:F)**	7:4	17:10	24:14
**Handedness (L:R)**	0:11	4:23	4:34
**NART**	116.9 (5.18)	118.6 (3.11)	116 (5.36)
**Chronicity (years)**	7 (5.8)	6.55 (4.44)	-
**Medical Atten. (%)**	81.8	66.1	-
**LOC (%)**	63.6	51.9	-
**Traffic Accid. (%)**	45.5	11.1	-
**Fall (%)**	27.3	37.0	-
**Assault (%)**	18.2	11.1	-
**Sport (%)**	9.1	29.6	-
**Indust. Accid. (%)**	0.0	3.7	-
**Home Accid. (%)**	0.0	7.4	-

**Table 2:** Pearson's Correlation Coefficient values are presented for all subtests (RT = Reaction Time and %E = Error) correlated with RPQ score. Significant values are highlighted with an asterisk.

*Subtest*		*PCS+*	*PCS-*	*Control*
	MOT	0.459*	-0.003	0.144
	BLC	0.746*	0.090	-0.084
**RT for CANTAB**	SWM	0.715*	0.264	-0.160
	IED	0.755*	0.171	0.248
	RTI	0.867*	0.860*	-0.067
	RVP	0.131	0.388	0.020
				
	ALRT	0.061	0.274	-0.050
	DA	0.872*	0.172	-0.007
**RT for TAP**	GNG	0.285	0.332	-0.084
	WM	0.749*	0.211	0.012
	CSNV	0.444	0.478	-0.010
	CSV	0.490	0.408	0.036
				
	MOT	-0.218	0.056	NS
	BLC	NS	-0.128	NS
**%E for CANTAB**	SWM	0.660*	0.385	-0.080
	IED	0.664*	0.230	0.307
	RTI	0.680*	0.132	0.019
	RVP	0.706*	0.615*	0.048
				
	ALRT	-0.187	-0.112	-0.214
	DA	0.570*	0.376	-0.058
**%E for TAP **	GN	0.085	0.291	-0.189
	WM	0.133	0.110	-0.112
	CSNV	-0.281	0.362	-0.135
	CSV	-0.003	-0.112	-0.226
